# Supplementary material for: Tailoring the resolution of single-cell RNA sequencing for primary cytotoxic T cells
Source: Nat Commun. 2021 Jan 25;12:569. doi: 10.1038/s41467-020-20751-7 (PMC7835213; doi:10.1038/s41467-020-20751-7)
Supplement: Supplementary file 1 — Supplementary Information [file 41467_2020_20751_MOESM1_ESM.pdf]

Supplementary Figure 1. Schematic protocol representation of SCRB-seq and Drop-seq.

(**A**) Schematic representation of the P14 experimental system. The green, blue and red colour denote naïve P14 T cells, P14 T cells recovered at day 8 post LCMV Armstrong infection and P14 T cells recovered at day 40 post LCMV clone-13 infection respectively. (**B**) Illustrates the difference in the single-cell capturing strategy of SCRB-seq versus Drop-seq. (**C**) Representation of the common chemistry downstream of single-cell capturing.

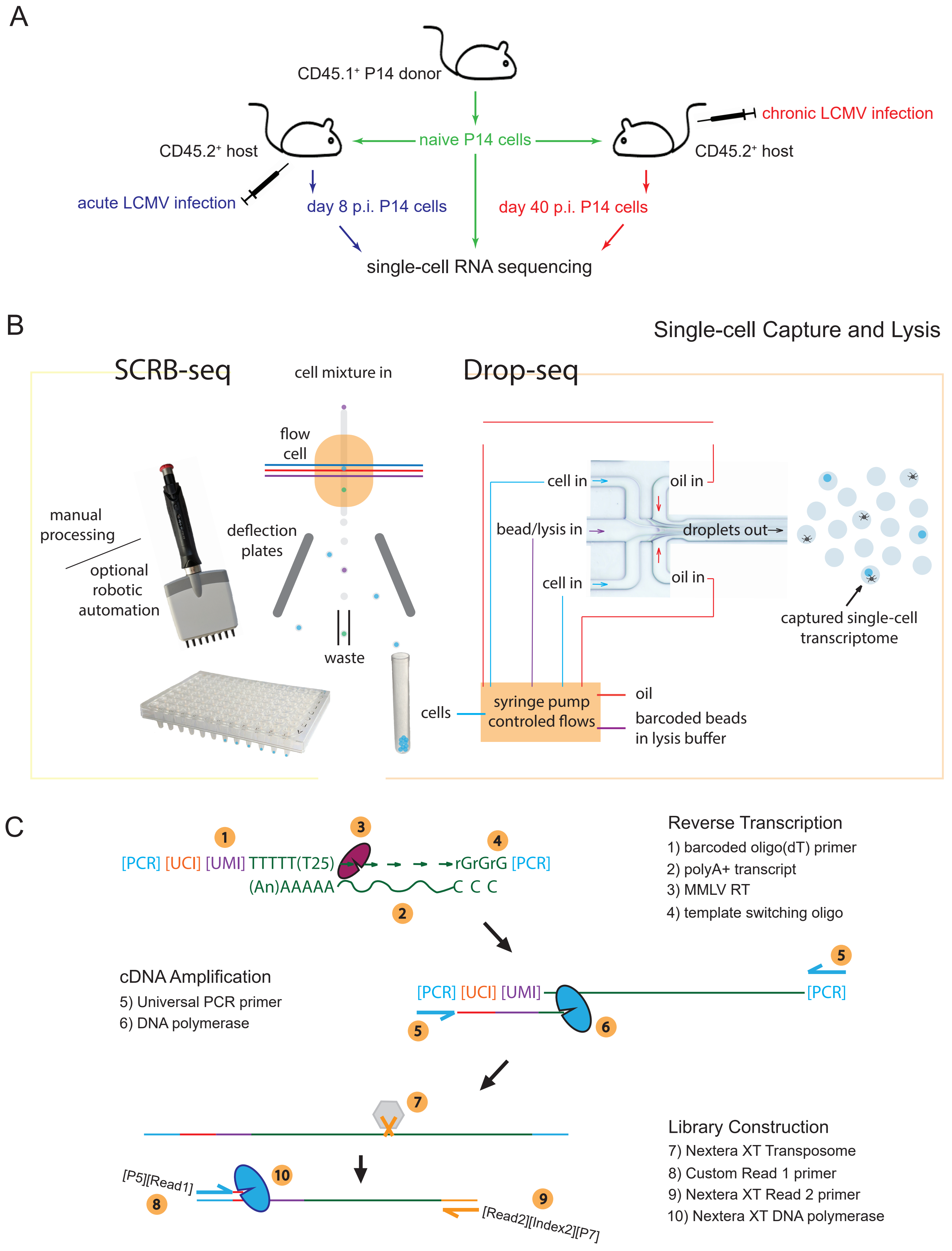

Supplementary Figure 2. Comparison of the cDNA yield among different Drop-seq optimizations.

(**A-C**) Bioanalyzer electropherograms comparing the amplified cDNA yield of Drop-seq between the original protocol and various optimizations.

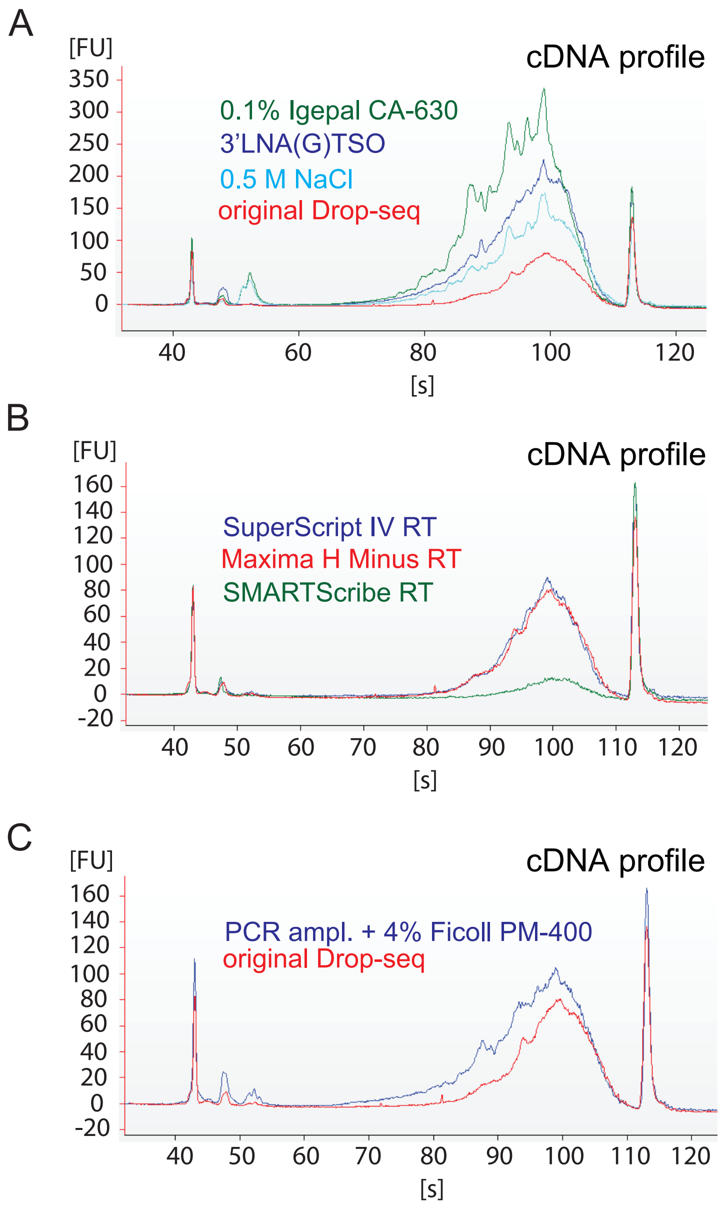

Supplementary Figure 3. Technical characteristics of libraries generated with tSCRB-seq and 10xGenomics Chromium from different cell types and states.

Comparative analysis of major technical parameters among a library generated with tSCBR-seq (P14 T cells recovered at day 8 post LCMV Armstrong infection) and published libraries generated with 10xGenomics Chromium (naïve or recovered at day 8 post LCMV Armstrong P14 T cells[22] and cord blood mononuclear cells available on the manufacturer's website).

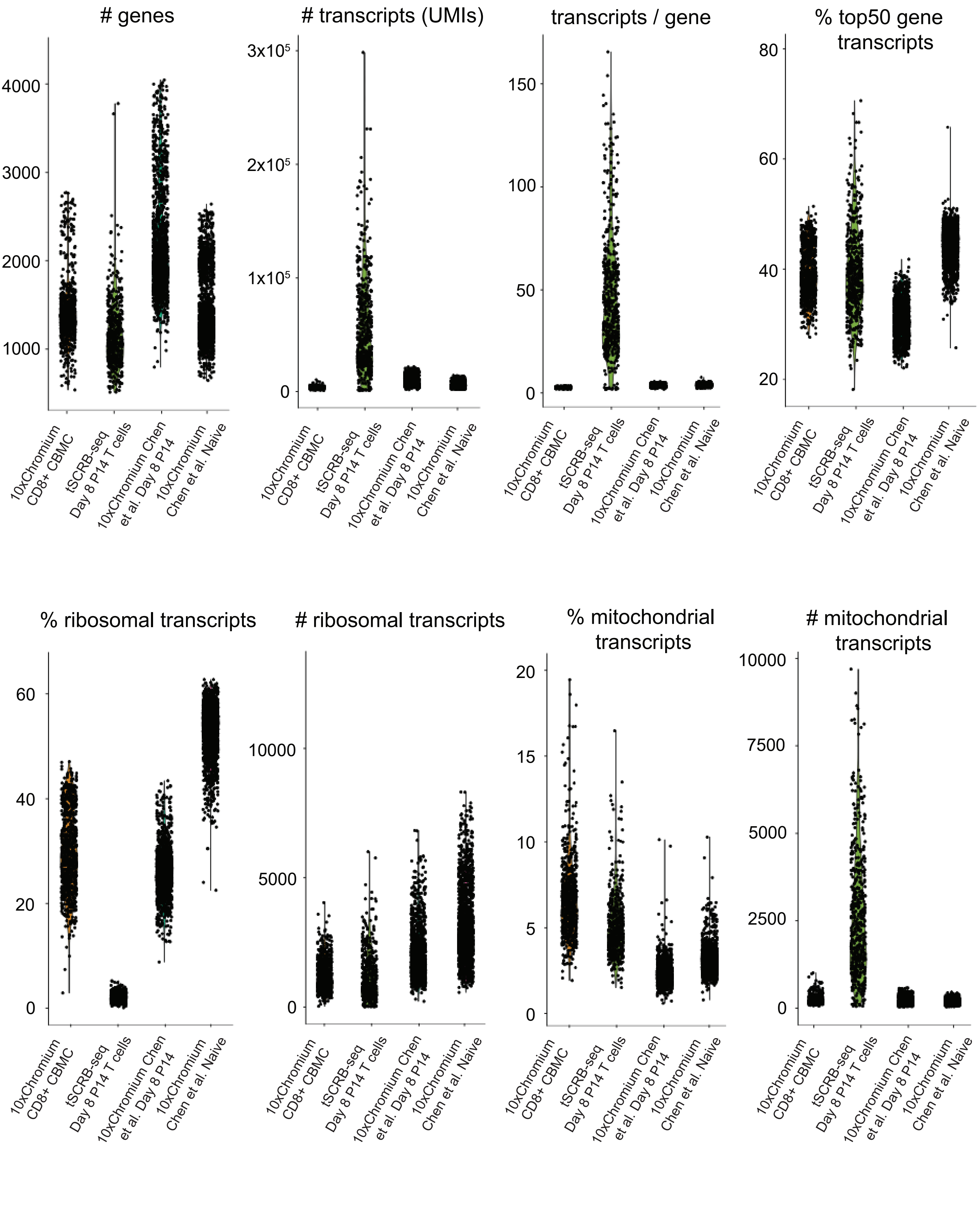

Supplementary Figure 4. The higher dynamic range of gene expression detected with tSCBR-seq results in improved compartment-resolved expression of key immune genes in chronic infection.

Comparative analysis of single-cell P14 T-cell transcriptomes generated with tSCRB-seq[12] or 10xChromium[13] and recovered from established LCMV clone-13 infections (on day 40 and day 30 respectively). Each circle represents a single-cell. The plots represent the expression of key for CD8 T cell differentiation and function immune genes and regulatory receptors depicted over tSNE. The expression level is illustrate using the red scale in plots based on the tSCBR-seq data and violet scale in plots using the 10xChromium data.

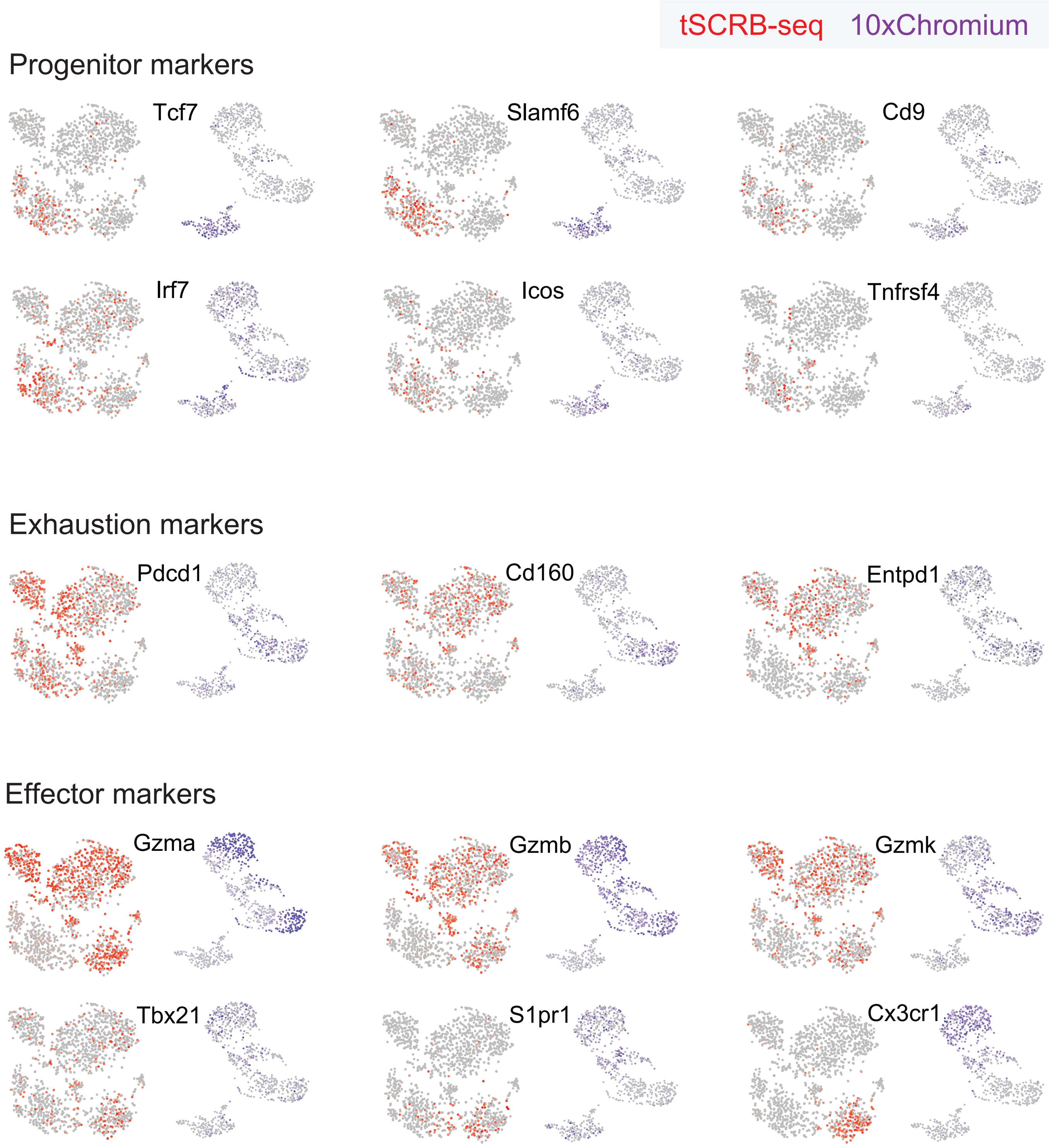

Supplementary Figure 5. Single-cell sort gating strategy.

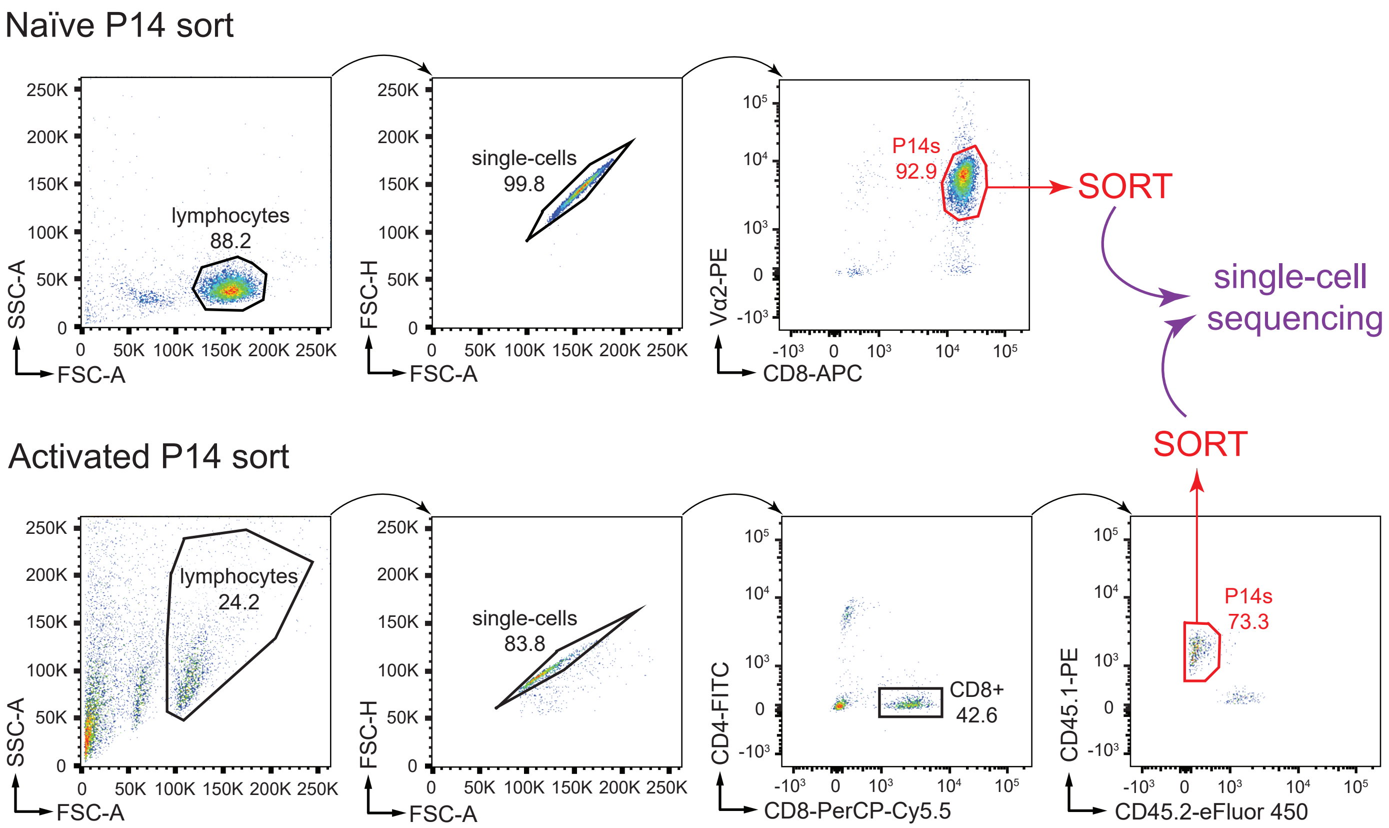

Supplementary Table 1. Fraction of cell positive for selected key immune genes among methods.

Analysis of libraries generated with tDrop-seq and tSCRB-seq from P14 T-cells recovered at day 8 post-acute LCMV Armstrong infection, compared to a published 10xChromium dataset with matching experimental setup[22]. Comparison of the fraction of cell detected to express key immune genes among methods.

P14 cells recovered on day 8 from acute infection			
GENE	tDROP-seq	tSCRB-seq	10xChromium
Gzma	87%	95%	42%
Gzmb	81%	89%	90%
Prf1	55%	64%	11%
Fasl	12%	10%	21%
Klrg1	67%	56%	55%
Id2	80%	76%	95%
Tbx21	38%	56%	47%
Klf2	72%	85%	43%
Klf3	46%	70%	54%
S1pr1	38%	41%	58%
S1pr4	40%	53%	92%
S1pr5	37%	42%	21%
Tcf7	10%	9%	24%
Slamf6	14%	12%	45%
Il7r	9%	6%	10%
Eomes	23%	6%	22%
Id3	0%	1%	5%
Bcl2	7%	9%	29%
Mki67	61%	31%	13%
Ezh2	35%	38%	29%
Cumulative detection	41%	42%	40%

Supplementary Table 2. Number of transcripts per cell of selected key immune genes detected by each method.

Comparative analysis of the number of transcripts per cell of key immune genes detected by tDrop-seq, tSCBR-seq and 10xGenomics Chromium[22] in P14 T cells recovered on day 8 post LCMV Armstrong infection. All sequencing data are down-sampled to 40 000 mapped reads per cell. Source data are provided as a Source Data file. The abbreviations are as follows: md. transcripts, median number of transcripts captured per cell; SD, standard deviation

P14 CD8 T cells recovered onday 8 post LCMV Armstrong infection						
	tDrop-seq		tSCRB-seq		10xChrom	
	md. # transcripts	SD	md. # transcripts	SD	md. # transcripts	SD
Gzma	7.0	17.5	180.0	405.1	4.0	34.7
Gzmb	3.0	3.7	62.0	115.0	9.0	11.9
Prf1	1.0	1.3	35.0	70.7	2.0	1.4
Fasl	1.0	0.7	23.0	46.3	1.0	0.5
Klrg1	2.0	2.3	22.0	34.8	2.0	2.1
Id2	4.0	4.0	35.5	72.8	11.0	10.0
Tbx21	1.0	0.6	20.0	32.9	1.0	1.0
Klf2	1.0	1.1	27.0	74.3	1.0	1.0
Klf3	2.0	1.8	28.5	43.8	1.0	1.2
S1pr1	1.0	0.9	14.0	24.2	1.0	1.1
S1pr4	1.0	0.9	13.0	18.5	3.0	2.8
S1pr5	1.5	1.3	26.0	55.6	1.0	1.0
Tcf7	2.0	0.8	12.5	28.5	1.0	1.9
Slamf6	2.0	1.0	23.5	46.7	1.0	1.8
Il7r	1.0	1.3	25.0	49.0	1.0	1.0
Eomes	1.0	1.5	6.5	42.6	1.0	0.8
Id3	1.0	0.0	9.0	37.5	1.0	1.8
Bcl2	2.0	0.6	12.0	20.3	1.0	1.6
Mki67	2.0	6.4	28.0	208.5	2.0	2.1
Ezh2	2.0	1.4	44.0	76.2	1.0	1.6

*All sequencing data were down-sampled to 40 000 mapped reads/cell.

Supplementary Table 3. Primer sequences.

Primer	Primer full name	Primer sequence	Producer	Purity	Note
DS-BEADS	ChemGenes Barcoded Oligo dT primer ON Beads	5'–Bead–Linker--TTTTTTTAAGCAGTGGTATCAAC GCAGAGTACJJJJJJJJJJJJNNNNNNNN TTTTTTTTTTTTTTTTTTTTTTTTTTTTTT--3'	ChemGenes Corporation	-	J denotes a nucleic acid part of the cellular barcode, which is identical in all primers attached to one bead; N denotes a random nucleic acid part of the unique molecular identifier, which is a unique sequence in each primar attached to one bead.
DS-TSO	Drop-seq Template Switching Oligo	AAGCAGTGGTATCAACGCAGAGTGAATrGrGrG	Eurogentec	HPLC	rG denotes a riboguanine
DS-TSO-LNA	Drop-seq Template Switching Oligo with Locked Nucleic Acid	AAGCAGTGGTATCAACGCAGAGTGAATrGrG+G	Eurogentec	HPLC	rG denotes a riboguanine; +G denotes a locked nucleic acid guanine
DS-SMART PCR	Drop-seq SMART PCR Primer	AAGCAGTGGTATCAACGCAGAGT	Integrated DNA Technologies	HPLC	
DS-P5	Drop-seq N5 primer	AATGATACGGCGACCACCGAGATCTACACGCCTGTCCGC GGAAGCAGTGGTATCAACGCAGAGT*A*C	Integrated DNA Technologies	HPLC	* denotes a phosphorothioate bond
DS-CR1	Drop-seq Custrom Read 1 Primer	GCCTGTCCGCGGAAGCAGTGGTATCAACGCAGAGTAC	Integrated DNA Technologies	HPLC	
SCRB-dT plate v1	tSCRB Barcoded Oligo-dT Primer Plate v1	/5Biosg/ACACTCTTTCCCTACACGACGCTCTTCCGATCTJ(6)N(10)T(30)VN	Integrated DNA Technologies	Ultramer Plate; Standard desalting	/5Biosg/ denotes a 5' biotin; J denotes a nucleic acid part of the cellular barcode; N denotes a random nucleic acid part of the unique molecular identifier; V denotes A or C or G
SCRB-dT plate v2	tSCRB Barcoded Oligo-dT Primer Plate v2 and v3	/5Biosg/ACACTCTTTCCCTACACGACGCTCTTCCGATCTJ(7)N(9)T(30)VN	Integrated DNA Technologies	Ultramer Plate; Standard desalting	/5Biosg/ denotes a 5' biotin; J denotes a nucleic acid part of the cellular barcode; N denotes a random nucleic acid part of the unique molecular identifier; V denotes A or C or G
SCRB-TSO	tSCRB-seq Template Switching Oligo	iC-iG-iCACACTCTTTCCCTACACGACGCrGrGrG	Eurogentec	HPLC	iC denotes a iso-dC; iG denotes a iso-dG; rG denotes a riboguanine
SCRB-TSO-LNA	SCRB-seq Template Switching Oligo with Locked Nucleic Acid	iC-iG-iCACACTCTTTCCCTACACGACGCrGrG+G	Eurogentec	HPLC	iC denotes a iso-dC; iG denotes a iso-dG; rG denotes a riboguanine; +G denotes a locked nucleic acid guanine
SCRB-SMART-PCR	SCRB-seq SMART PCR Primer	/5Biosg/ACACTCTTTCCCTACACGACGC	Integrated DNA Technologies	HPLC	/5Biosg/ denotes a 5' biotin
SMART-P5	SCRB-seq N5 primer	AATGATACGGCGACCACCGAGATCTACACTCTTTCCCTACACGACGC TCTTCCG*A*T*C*T*	Integrated DNA Technologies	HPLC	* denotes a phosphorothioate bond
SCRB-CR1	SCRB-seq Custrom Read 1 Primer	TCTTTCCCTACACGACGCTCTTCCGATCT	Integrated DNA Technologies	HPLC	

Supplementary Table 4. tSCRB Barcoded Oligo-dT Primer Plate v1

/5Biosg/ACACTCTTTCCCTACACGACGCTCTTCCGATCTJ(6)N(10)T(30)VN

/5Biosg/ denotes a 5' biotin; J denotes a nucleic acid part of the cellular barcode; N denotes a random nucleic acid part of the unique molecular identifier; V denotes A or C or G; IDT Ultramer Plate; Standard desalting

A1	/5Biosg/ACACTCTTTCCCTACACGACGCTCTTCCGATCTAAACGCNNNNNNNNNNTTTTTTTTTTTTTTTTTTTTTTTTTTTTTTVN	E1	/5Biosg/ACACTCTTTCCCTACACGACGCTCTTCCGATCTATGCTCNNNNNNNNNNTTTTTTTTTTTTTTTTTTTTTTTTTTTTTTVN
A2	/5Biosg/ACACTCTTTCCCTACACGACGCTCTTCCGATCTAACACGNNNNNNNNNNTTTTTTTTTTTTTTTTTTTTTTTTTTTTTTVN	E2	/5Biosg/ACACTCTTTCCCTACACGACGCTCTTCCGATCTATGGTGNNNNNNNNNNTTTTTTTTTTTTTTTTTTTTTTTTTTTTTTVN
A3	/5Biosg/ACACTCTTTCCCTACACGACGCTCTTCCGATCTAACCAGNNNNNNNNNNTTTTTTTTTTTTTTTTTTTTTTTTTTTTTTVN	E3	/5Biosg/ACACTCTTTCCCTACACGACGCTCTTCCGATCTATTCCGNNNNNNNNNNTTTTTTTTTTTTTTTTTTTTTTTTTTTTTTVN
A4	/5Biosg/ACACTCTTTCCCTACACGACGCTCTTCCGATCTAACCGTNNNNNNNNNNTTTTTTTTTTTTTTTTTTTTTTTTTTTTTTVN	E4	/5Biosg/ACACTCTTTCCCTACACGACGCTCTTCCGATCTATTGCCNNNNNNNNNNTTTTTTTTTTTTTTTTTTTTTTTTTTTTTTVN
A5	/5Biosg/ACACTCTTTCCCTACACGACGCTCTTCCGATCTAACGTGNNNNNNNNNNTTTTTTTTTTTTTTTTTTTTTTTTTTTTTTVN	E5	/5Biosg/ACACTCTTTCCCTACACGACGCTCTTCCGATCTCAACACNNNNNNNNNNTTTTTTTTTTTTTTTTTTTTTTTTTTTTTTVN
A6	/5Biosg/ACACTCTTTCCCTACACGACGCTCTTCCGATCTAACTGGNNNNNNNNNNTTTTTTTTTTTTTTTTTTTTTTTTTTTTTTVN	E6	/5Biosg/ACACTCTTTCCCTACACGACGCTCTTCCGATCTCAACGTNNNNNNNNNNTTTTTTTTTTTTTTTTTTTTTTTTTTTTTTVN
A7	/5Biosg/ACACTCTTTCCCTACACGACGCTCTTCCGATCTAAGCCTNNNNNNNNNNTTTTTTTTTTTTTTTTTTTTTTTTTTTTTTVN	E7	/5Biosg/ACACTCTTTCCCTACACGACGCTCTTCCGATCTCAAGAGNNNNNNNNNNTTTTTTTTTTTTTTTTTTTTTTTTTTTTTTVN
A8	/5Biosg/ACACTCTTTCCCTACACGACGCTCTTCCGATCTAAGGACNNNNNNNNNNTTTTTTTTTTTTTTTTTTTTTTTTTTTTTTVN	E8	/5Biosg/ACACTCTTTCCCTACACGACGCTCTTCCGATCTCAAGTCNNNNNNNNNNTTTTTTTTTTTTTTTTTTTTTTTTTTTTTTVN
A9	/5Biosg/ACACTCTTTCCCTACACGACGCTCTTCCGATCTAAGTCGNNNNNNNNNNTTTTTTTTTTTTTTTTTTTTTTTTTTTTTTVN	E9	/5Biosg/ACACTCTTTCCCTACACGACGCTCTTCCGATCTCACAACNNNNNNNNNNTTTTTTTTTTTTTTTTTTTTTTTTTTTTTTVN
A10	/5Biosg/ACACTCTTTCCCTACACGACGCTCTTCCGATCTAATCGGNNNNNNNNNNTTTTTTTTTTTTTTTTTTTTTTTTTTTTTTVN	E10	/5Biosg/ACACTCTTTCCCTACACGACGCTCTTCCGATCTCACGTTNNNNNNNNNNTTTTTTTTTTTTTTTTTTTTTTTTTTTTTTVN
A11	/5Biosg/ACACTCTTTCCCTACACGACGCTCTTCCGATCTACAAGCNNNNNNNNNNTTTTTTTTTTTTTTTTTTTTTTTTTTTTTTVN	E11	/5Biosg/ACACTCTTTCCCTACACGACGCTCTTCCGATCTCACTTGNNNNNNNNNNTTTTTTTTTTTTTTTTTTTTTTTTTTTTTTVN
A12	/5Biosg/ACACTCTTTCCCTACACGACGCTCTTCCGATCTACACGTNNNNNNNNNNTTTTTTTTTTTTTTTTTTTTTTTTTTTTTTVN	E12	/5Biosg/ACACTCTTTCCCTACACGACGCTCTTCCGATCTCAGATCNNNNNNNNNNTTTTTTTTTTTTTTTTTTTTTTTTTTTTTTVN
B1	/5Biosg/ACACTCTTTCCCTACACGACGCTCTTCCGATCTACAGACNNNNNNNNNNTTTTTTTTTTTTTTTTTTTTTTTTTTTTTTVN	F1	/5Biosg/ACACTCTTTCCCTACACGACGCTCTTCCGATCTCAGGTANNNNNNNNNNTTTTTTTTTTTTTTTTTTTTTTTTTTTTTTVN
B2	/5Biosg/ACACTCTTTCCCTACACGACGCTCTTCCGATCTACAGTGNNNNNNNNNNTTTTTTTTTTTTTTTTTTTTTTTTTTTTTTVN	F2	/5Biosg/ACACTCTTTCCCTACACGACGCTCTTCCGATCTCAGTGTNNNNNNNNNNTTTTTTTTTTTTTTTTTTTTTTTTTTTTTTVN
B3	/5Biosg/ACACTCTTTCCCTACACGACGCTCTTCCGATCTACCAAGNNNNNNNNNNTTTTTTTTTTTTTTTTTTTTTTTTTTTTTTVN	F3	/5Biosg/ACACTCTTTCCCTACACGACGCTCTTCCGATCTCATAGCNNNNNNNNNNTTTTTTTTTTTTTTTTTTTTTTTTTTTTTTVN
B4	/5Biosg/ACACTCTTTCCCTACACGACGCTCTTCCGATCTACCAGANNNNNNNNNNTTTTTTTTTTTTTTTTTTTTTTTTTTTTTTVN	F4	/5Biosg/ACACTCTTTCCCTACACGACGCTCTTCCGATCTCATCGANNNNNNNNNNTTTTTTTTTTTTTTTTTTTTTTTTTTTTTTVN
B5	/5Biosg/ACACTCTTTCCCTACACGACGCTCTTCCGATCTACCTCANNNNNNNNNNTTTTTTTTTTTTTTTTTTTTTTTTTTTTTTVN	F5	/5Biosg/ACACTCTTTCCCTACACGACGCTCTTCCGATCTCATGACNNNNNNNNNNTTTTTTTTTTTTTTTTTTTTTTTTTTTTTTVN
B6	/5Biosg/ACACTCTTTCCCTACACGACGCTCTTCCGATCTACCTTCNNNNNNNNNNTTTTTTTTTTTTTTTTTTTTTTTTTTTTTTVN	F6	/5Biosg/ACACTCTTTCCCTACACGACGCTCTTCCGATCTCATGTGNNNNNNNNNNTTTTTTTTTTTTTTTTTTTTTTTTTTTTTTVN
B7	/5Biosg/ACACTCTTTCCCTACACGACGCTCTTCCGATCTACGACANNNNNNNNNNTTTTTTTTTTTTTTTTTTTTTTTTTTTTTTVN	F7	/5Biosg/ACACTCTTTCCCTACACGACGCTCTTCCGATCTCCAAGANNNNNNNNNNTTTTTTTTTTTTTTTTTTTTTTTTTTTTTTVN
B8	/5Biosg/ACACTCTTTCCCTACACGACGCTCTTCCGATCTACGATGNNNNNNNNNNTTTTTTTTTTTTTTTTTTTTTTTTTTTTTTVN	F8	/5Biosg/ACACTCTTTCCCTACACGACGCTCTTCCGATCTCCAGTANNNNNNNNNNTTTTTTTTTTTTTTTTTTTTTTTTTTTTTTVN
B9	/5Biosg/ACACTCTTTCCCTACACGACGCTCTTCCGATCTACGCTANNNNNNNNNNTTTTTTTTTTTTTTTTTTTTTTTTTTTTTTVN	F9	/5Biosg/ACACTCTTTCCCTACACGACGCTCTTCCGATCTCCATTCNNNNNNNNNNTTTTTTTTTTTTTTTTTTTTTTTTTTTTTTVN
B10	/5Biosg/ACACTCTTTCCCTACACGACGCTCTTCCGATCTACGTCTNNNNNNNNNNTTTTTTTTTTTTTTTTTTTTTTTTTTTTTTVN	F10	/5Biosg/ACACTCTTTCCCTACACGACGCTCTTCCGATCTCCCATANNNNNNNNNNTTTTTTTTTTTTTTTTTTTTTTTTTTTTTTVN
B11	/5Biosg/ACACTCTTTCCCTACACGACGCTCTTCCGATCTACTACGNNNNNNNNNNTTTTTTTTTTTTTTTTTTTTTTTTTTTTTTVN	F11	/5Biosg/ACACTCTTTCCCTACACGACGCTCTTCCGATCTCCGAAANNNNNNNNNNTTTTTTTTTTTTTTTTTTTTTTTTTTTTTTVN
B12	/5Biosg/ACACTCTTTCCCTACACGACGCTCTTCCGATCTACTCCANNNNNNNNNNTTTTTTTTTTTTTTTTTTTTTTTTTTTTTTVN	F12	/5Biosg/ACACTCTTTCCCTACACGACGCTCTTCCGATCTCCGTTANNNNNNNNNNTTTTTTTTTTTTTTTTTTTTTTTTTTTTTTVN
C1	/5Biosg/ACACTCTTTCCCTACACGACGCTCTTCCGATCTACTGCTNNNNNNNNNNTTTTTTTTTTTTTTTTTTTTTTTTTTTTTTVN	G1	/5Biosg/ACACTCTTTCCCTACACGACGCTCTTCCGATCTCCTACANNNNNNNNNNTTTTTTTTTTTTTTTTTTTTTTTTTTTTTTVN
C2	/5Biosg/ACACTCTTTCCCTACACGACGCTCTTCCGATCTACTGTCNNNNNNNNNNTTTTTTTTTTTTTTTTTTTTTTTTTTTTTTVN	G2	/5Biosg/ACACTCTTTCCCTACACGACGCTCTTCCGATCTCCTGTTNNNNNNNNNNTTTTTTTTTTTTTTTTTTTTTTTTTTTTTTVN
C3	/5Biosg/ACACTCTTTCCCTACACGACGCTCTTCCGATCTAGAACCNNNNNNNNNNTTTTTTTTTTTTTTTTTTTTTTTTTTTTTTVN	G3	/5Biosg/ACACTCTTTCCCTACACGACGCTCTTCCGATCTCGAAAGNNNNNNNNNNTTTTTTTTTTTTTTTTTTTTTTTTTTTTTTVN
C4	/5Biosg/ACACTCTTTCCCTACACGACGCTCTTCCGATCTAGACGANNNNNNNNNNTTTTTTTTTTTTTTTTTTTTTTTTTTTTTTVN	G4	/5Biosg/ACACTCTTTCCCTACACGACGCTCTTCCGATCTCGAATCNNNNNNNNNNTTTTTTTTTTTTTTTTTTTTTTTTTTTTTTVN
C5	/5Biosg/ACACTCTTTCCCTACACGACGCTCTTCCGATCTAGAGAGNNNNNNNNNNTTTTTTTTTTTTTTTTTTTTTTTTTTTTTTVN	G5	/5Biosg/ACACTCTTTCCCTACACGACGCTCTTCCGATCTCGAGATNNNNNNNNNNTTTTTTTTTTTTTTTTTTTTTTTTTTTTTTVN
C6	/5Biosg/ACACTCTTTCCCTACACGACGCTCTTCCGATCTAGAGTCNNNNNNNNNNTTTTTTTTTTTTTTTTTTTTTTTTTTTTTTVN	G6	/5Biosg/ACACTCTTTCCCTACACGACGCTCTTCCGATCTCGATCANNNNNNNNNNTTTTTTTTTTTTTTTTTTTTTTTTTTTTTTVN
C7	/5Biosg/ACACTCTTTCCCTACACGACGCTCTTCCGATCTAGCAGTNNNNNNNNNNTTTTTTTTTTTTTTTTTTTTTTTTTTTTTTVN	G7	/5Biosg/ACACTCTTTCCCTACACGACGCTCTTCCGATCTCGCTAANNNNNNNNNNTTTTTTTTTTTTTTTTTTTTTTTTTTTTTTVN
C8	/5Biosg/ACACTCTTTCCCTACACGACGCTCTTCCGATCTAGCCATNNNNNNNNNNTTTTTTTTTTTTTTTTTTTTTTTTTTTTTTVN	G8	/5Biosg/ACACTCTTTCCCTACACGACGCTCTTCCGATCTCGGATANNNNNNNNNNTTTTTTTTTTTTTTTTTTTTTTTTTTTTTTVN
C9	/5Biosg/ACACTCTTTCCCTACACGACGCTCTTCCGATCTAGCGAANNNNNNNNNNTTTTTTTTTTTTTTTTTTTTTTTTTTTTTTVN	G9	/5Biosg/ACACTCTTTCCCTACACGACGCTCTTCCGATCTCGTAGTNNNNNNNNNNTTTTTTTTTTTTTTTTTTTTTTTTTTTTTTVN
C10	/5Biosg/ACACTCTTTCCCTACACGACGCTCTTCCGATCTAGCTACNNNNNNNNNNTTTTTTTTTTTTTTTTTTTTTTTTTTTTTTVN	G10	/5Biosg/ACACTCTTTCCCTACACGACGCTCTTCCGATCTCGTCATNNNNNNNNNNTTTTTTTTTTTTTTTTTTTTTTTTTTTTTTVN
C11	/5Biosg/ACACTCTTTCCCTACACGACGCTCTTCCGATCTAGCTGANNNNNNNNNNTTTTTTTTTTTTTTTTTTTTTTTTTTTTTTVN	G11	/5Biosg/ACACTCTTTCCCTACACGACGCTCTTCCGATCTCGTTGANNNNNNNNNNTTTTTTTTTTTTTTTTTTTTTTTTTTTTTTVN
C12	/5Biosg/ACACTCTTTCCCTACACGACGCTCTTCCGATCTAGGACTNNNNNNNNNNTTTTTTTTTTTTTTTTTTTTTTTTTTTTTTVN	G12	/5Biosg/ACACTCTTTCCCTACACGACGCTCTTCCGATCTCTAACGNNNNNNNNNNTTTTTTTTTTTTTTTTTTTTTTTTTTTTTTVN
D1	/5Biosg/ACACTCTTTCCCTACACGACGCTCTTCCGATCTAGGCTTNNNNNNNNNNTTTTTTTTTTTTTTTTTTTTTTTTTTTTTTVN	H1	/5Biosg/ACACTCTTTCCCTACACGACGCTCTTCCGATCTCTACAGNNNNNNNNNNTTTTTTTTTTTTTTTTTTTTTTTTTTTTTTVN
D2	/5Biosg/ACACTCTTTCCCTACACGACGCTCTTCCGATCTAGGTAGNNNNNNNNNNTTTTTTTTTTTTTTTTTTTTTTTTTTTTTTVN	H2	/5Biosg/ACACTCTTTCCCTACACGACGCTCTTCCGATCTCTACGANNNNNNNNNNTTTTTTTTTTTTTTTTTTTTTTTTTTTTTTVN
D3	/5Biosg/ACACTCTTTCCCTACACGACGCTCTTCCGATCTAGGTGTNNNNNNNNNNTTTTTTTTTTTTTTTTTTTTTTTTTTTTTTVN	H3	/5Biosg/ACACTCTTTCCCTACACGACGCTCTTCCGATCTCTAGACNNNNNNNNNNTTTTTTTTTTTTTTTTTTTTTTTTTTTTTTVN
D4	/5Biosg/ACACTCTTTCCCTACACGACGCTCTTCCGATCTAGTCAGNNNNNNNNNNTTTTTTTTTTTTTTTTTTTTTTTTTTTTTTVN	H4	/5Biosg/ACACTCTTTCCCTACACGACGCTCTTCCGATCTCTATGGNNNNNNNNNNTTTTTTTTTTTTTTTTTTTTTTTTTTTTTTVN
D5	/5Biosg/ACACTCTTTCCCTACACGACGCTCTTCCGATCTAGTCTCNNNNNNNNNNTTTTTTTTTTTTTTTTTTTTTTTTTTTTTTVN	H5	/5Biosg/ACACTCTTTCCCTACACGACGCTCTTCCGATCTCTCATCNNNNNNNNNNTTTTTTTTTTTTTTTTTTTTTTTTTTTTTTVN
D6	/5Biosg/ACACTCTTTCCCTACACGACGCTCTTCCGATCTAGTGCANNNNNNNNNNTTTTTTTTTTTTTTTTTTTTTTTTTTTTTTVN	H6	/5Biosg/ACACTCTTTCCCTACACGACGCTCTTCCGATCTCTCGATNNNNNNNNNNTTTTTTTTTTTTTTTTTTTTTTTTTTTTTTVN
D7	/5Biosg/ACACTCTTTCCCTACACGACGCTCTTCCGATCTAGTTCCNNNNNNNNNNTTTTTTTTTTTTTTTTTTTTTTTTTTTTTTVN	H7	/5Biosg/ACACTCTTTCCCTACACGACGCTCTTCCGATCTCTCTGTNNNNNNNNNNTTTTTTTTTTTTTTTTTTTTTTTTTTTTTTVN
D8	/5Biosg/ACACTCTTTCCCTACACGACGCTCTTCCGATCTATAGGCNNNNNNNNNNTTTTTTTTTTTTTTTTTTTTTTTTTTTTTTVN	H8	/5Biosg/ACACTCTTTCCCTACACGACGCTCTTCCGATCTCTGAGANNNNNNNNNNTTTTTTTTTTTTTTTTTTTTTTTTTTTTTTVN
D9	/5Biosg/ACACTCTTTCCCTACACGACGCTCTTCCGATCTATCCACNNNNNNNNNNTTTTTTTTTTTTTTTTTTTTTTTTTTTTTTVN	H9	/5Biosg/ACACTCTTTCCCTACACGACGCTCTTCCGATCTCTGGAANNNNNNNNNNTTTTTTTTTTTTTTTTTTTTTTTTTTTTTTVN
D10	/5Biosg/ACACTCTTTCCCTACACGACGCTCTTCCGATCTATCCTGNNNNNNNNNNTTTTTTTTTTTTTTTTTTTTTTTTTTTTTTVN	H10	/5Biosg/ACACTCTTTCCCTACACGACGCTCTTCCGATCTCTGTCTNNNNNNNNNNTTTTTTTTTTTTTTTTTTTTTTTTTTTTTTVN
D11	/5Biosg/ACACTCTTTCCCTACACGACGCTCTTCCGATCTATCGTCNNNNNNNNNNTTTTTTTTTTTTTTTTTTTTTTTTTTTTTTVN	H11	/5Biosg/ACACTCTTTCCCTACACGACGCTCTTCCGATCTCTTACCNNNNNNNNNNTTTTTTTTTTTTTTTTTTTTTTTTTTTTTTVN
D12	/5Biosg/ACACTCTTTCCCTACACGACGCTCTTCCGATCTATGACCNNNNNNNNNNTTTTTTTTTTTTTTTTTTTTTTTTTTTTTTVN	H12	/5Biosg/ACACTCTTTCCCTACACGACGCTCTTCCGATCTCTTCACNNNNNNNNNNTTTTTTTTTTTTTTTTTTTTTTTTTTTTTTVN

Supplementary Table 5. tSCRB Barcoded Oligo-dT Primer Plate v2

/5Biosg/ACACTCTTTCCCTACACGACGCTCTTCCGATCTJ(7)N(9)T(30)VN

/5Biosg/ denotes a 5' biotin; J denotes a nucleic acid part of the cellular barcode; N denotes a random nucleic acid part of the unique molecular identifier; V denotes A or C or G; IDT Ultramer Plate; Standard desalting

A1	/5Biosg/ACACTCTTTCCCTACACGACGCTCTTCCGATCTGGCTTTCNNNNNNNNNTTTTTTTTTTTTTTTTTTTTTTTTTTTTTTVN	E1	/5Biosg/ACACTCTTTCCCTACACGACGCTCTTCCGATCTAGGTCAGNNNNNNNNNTTTTTTTTTTTTTTTTTTTTTTTTTTTTTTVN
A2	/5Biosg/ACACTCTTTCCCTACACGACGCTCTTCCGATCTCATCTGCNNNNNNNNNTTTTTTTTTTTTTTTTTTTTTTTTTTTTTTVN	E2	/5Biosg/ACACTCTTTCCCTACACGACGCTCTTCCGATCTCTACCGANNNNNNNNNTTTTTTTTTTTTTTTTTTTTTTTTTTTTTTVN
A3	/5Biosg/ACACTCTTTCCCTACACGACGCTCTTCCGATCTAGTCACANNNNNNNNNTTTTTTTTTTTTTTTTTTTTTTTTTTTTTTVN	E3	/5Biosg/ACACTCTTTCCCTACACGACGCTCTTCCGATCTTGTTCTGNNNNNNNNNTTTTTTTTTTTTTTTTTTTTTTTTTTTTTTVN
A4	/5Biosg/ACACTCTTTCCCTACACGACGCTCTTCCGATCTGCTGCTTNNNNNNNNNTTTTTTTTTTTTTTTTTTTTTTTTTTTTTTVN	E4	/5Biosg/ACACTCTTTCCCTACACGACGCTCTTCCGATCTACCATTCNNNNNNNNNTTTTTTTTTTTTTTTTTTTTTTTTTTTTTTVN
A5	/5Biosg/ACACTCTTTCCCTACACGACGCTCTTCCGATCTACTGTTGNNNNNNNNNTTTTTTTTTTTTTTTTTTTTTTTTTTTTTTVN	E5	/5Biosg/ACACTCTTTCCCTACACGACGCTCTTCCGATCTCCCTCTTNNNNNNNNNTTTTTTTTTTTTTTTTTTTTTTTTTTTTTTVN
A6	/5Biosg/ACACTCTTTCCCTACACGACGCTCTTCCGATCTACACTGANNNNNNNNNTTTTTTTTTTTTTTTTTTTTTTTTTTTTTTVN	E6	/5Biosg/ACACTCTTTCCCTACACGACGCTCTTCCGATCTGGACACTNNNNNNNNNTTTTTTTTTTTTTTTTTTTTTTTTTTTTTTVN
A7	/5Biosg/ACACTCTTTCCCTACACGACGCTCTTCCGATCTTGACGAANNNNNNNNNTTTTTTTTTTTTTTTTTTTTTTTTTTTTTTVN	E7	/5Biosg/ACACTCTTTCCCTACACGACGCTCTTCCGATCTATAGCCGNNNNNNNNNTTTTTTTTTTTTTTTTTTTTTTTTTTTTTTVN
A8	/5Biosg/ACACTCTTTCCCTACACGACGCTCTTCCGATCTTGCACGTNNNNNNNNNTTTTTTTTTTTTTTTTTTTTTTTTTTTTTTVN	E8	/5Biosg/ACACTCTTTCCCTACACGACGCTCTTCCGATCTAGTTCGCNNNNNNNNNTTTTTTTTTTTTTTTTTTTTTTTTTTTTTTVN
A9	/5Biosg/ACACTCTTTCCCTACACGACGCTCTTCCGATCTCACAAGCNNNNNNNNNTTTTTTTTTTTTTTTTTTTTTTTTTTTTTTVN	E9	/5Biosg/ACACTCTTTCCCTACACGACGCTCTTCCGATCTGAGTAACNNNNNNNNNTTTTTTTTTTTTTTTTTTTTTTTTTTTTTTVN
A10	/5Biosg/ACACTCTTTCCCTACACGACGCTCTTCCGATCTGTATGACNNNNNNNNNTTTTTTTTTTTTTTTTTTTTTTTTTTTTTTVN	E10	/5Biosg/ACACTCTTTCCCTACACGACGCTCTTCCGATCTCCGATAGNNNNNNNNNTTTTTTTTTTTTTTTTTTTTTTTTTTTTTTVN
A11	/5Biosg/ACACTCTTTCCCTACACGACGCTCTTCCGATCTAGAGCTCNNNNNNNNNTTTTTTTTTTTTTTTTTTTTTTTTTTTTTTVN	E11	/5Biosg/ACACTCTTTCCCTACACGACGCTCTTCCGATCTTGAAGCCNNNNNNNNNTTTTTTTTTTTTTTTTTTTTTTTTTTTTTTVN
A12	/5Biosg/ACACTCTTTCCCTACACGACGCTCTTCCGATCTTGGGTGTNNNNNNNNNTTTTTTTTTTTTTTTTTTTTTTTTTTTTTTVN	E12	/5Biosg/ACACTCTTTCCCTACACGACGCTCTTCCGATCTTTCCGACNNNNNNNNNTTTTTTTTTTTTTTTTTTTTTTTTTTTTTTVN
B1	/5Biosg/ACACTCTTTCCCTACACGACGCTCTTCCGATCTAAATGCGNNNNNNNNNTTTTTTTTTTTTTTTTTTTTTTTTTTTTTTVN	F1	/5Biosg/ACACTCTTTCCCTACACGACGCTCTTCCGATCTCAAGACTNNNNNNNNNTTTTTTTTTTTTTTTTTTTTTTTTTTTTTTVN
B2	/5Biosg/ACACTCTTTCCCTACACGACGCTCTTCCGATCTGAAGCGTNNNNNNNNNTTTTTTTTTTTTTTTTTTTTTTTTTTTTTTVN	F2	/5Biosg/ACACTCTTTCCCTACACGACGCTCTTCCGATCTACGGGATNNNNNNNNNTTTTTTTTTTTTTTTTTTTTTTTTTTTTTTVN
B3	/5Biosg/ACACTCTTTCCCTACACGACGCTCTTCCGATCTGCCATATNNNNNNNNNTTTTTTTTTTTTTTTTTTTTTTTTTTTTTTVN	F3	/5Biosg/ACACTCTTTCCCTACACGACGCTCTTCCGATCTAACGGTTNNNNNNNNNTTTTTTTTTTTTTTTTTTTTTTTTTTTTTTVN
B4	/5Biosg/ACACTCTTTCCCTACACGACGCTCTTCCGATCTATGCATGNNNNNNNNNTTTTTTTTTTTTTTTTTTTTTTTTTTTTTTVN	F4	/5Biosg/ACACTCTTTCCCTACACGACGCTCTTCCGATCTTACGCCTNNNNNNNNNTTTTTTTTTTTTTTTTTTTTTTTTTTTTTTVN
B5	/5Biosg/ACACTCTTTCCCTACACGACGCTCTTCCGATCTTCGGACANNNNNNNNNTTTTTTTTTTTTTTTTTTTTTTTTTTTTTTVN	F5	/5Biosg/ACACTCTTTCCCTACACGACGCTCTTCCGATCTCACCATGNNNNNNNNNTTTTTTTTTTTTTTTTTTTTTTTTTTTTTTVN
B6	/5Biosg/ACACTCTTTCCCTACACGACGCTCTTCCGATCTGATCCACNNNNNNNNNTTTTTTTTTTTTTTTTTTTTTTTTTTTTTTVN	F6	/5Biosg/ACACTCTTTCCCTACACGACGCTCTTCCGATCTCGAGGTTNNNNNNNNNTTTTTTTTTTTTTTTTTTTTTTTTTTTTTTVN
B7	/5Biosg/ACACTCTTTCCCTACACGACGCTCTTCCGATCTCGGTATANNNNNNNNNTTTTTTTTTTTTTTTTTTTTTTTTTTTTTTVN	F7	/5Biosg/ACACTCTTTCCCTACACGACGCTCTTCCGATCTTTCTGGANNNNNNNNNTTTTTTTTTTTTTTTTTTTTTTTTTTTTTTVN
B8	/5Biosg/ACACTCTTTCCCTACACGACGCTCTTCCGATCTACAGGCANNNNNNNNNTTTTTTTTTTTTTTTTTTTTTTTTTTTTTTVN	F8	/5Biosg/ACACTCTTTCCCTACACGACGCTCTTCCGATCTGGATTCGNNNNNNNNNTTTTTTTTTTTTTTTTTTTTTTTTTTTTTTVN
B9	/5Biosg/ACACTCTTTCCCTACACGACGCTCTTCCGATCTGTAATGGNNNNNNNNNTTTTTTTTTTTTTTTTTTTTTTTTTTTTTTVN	F9	/5Biosg/ACACTCTTTCCCTACACGACGCTCTTCCGATCTGTTTGCGNNNNNNNNNTTTTTTTTTTTTTTTTTTTTTTTTTTTTTTVN
B10	/5Biosg/ACACTCTTTCCCTACACGACGCTCTTCCGATCTAGGCTTANNNNNNNNNTTTTTTTTTTTTTTTTTTTTTTTTTTTTTTVN	F10	/5Biosg/ACACTCTTTCCCTACACGACGCTCTTCCGATCTCCACACANNNNNNNNNTTTTTTTTTTTTTTTTTTTTTTTTTTTTTTVN
B11	/5Biosg/ACACTCTTTCCCTACACGACGCTCTTCCGATCTGGGCAAANNNNNNNNNTTTTTTTTTTTTTTTTTTTTTTTTTTTTTTVN	F11	/5Biosg/ACACTCTTTCCCTACACGACGCTCTTCCGATCTCCTTTGGNNNNNNNNNTTTTTTTTTTTTTTTTTTTTTTTTTTTTTTVN
B12	/5Biosg/ACACTCTTTCCCTACACGACGCTCTTCCGATCTCTGAGACNNNNNNNNNTTTTTTTTTTTTTTTTTTTTTTTTTTTTTTVN	F12	/5Biosg/ACACTCTTTCCCTACACGACGCTCTTCCGATCTACTACGTNNNNNNNNNTTTTTTTTTTTTTTTTTTTTTTTTTTTTTTVN
C1	/5Biosg/ACACTCTTTCCCTACACGACGCTCTTCCGATCTATACACCNNNNNNNNNTTTTTTTTTTTTTTTTTTTTTTTTTTTTTTVN	G1	/5Biosg/ACACTCTTTCCCTACACGACGCTCTTCCGATCTTCAACTCNNNNNNNNNTTTTTTTTTTTTTTTTTTTTTTTTTTTTTTVN
C2	/5Biosg/ACACTCTTTCCCTACACGACGCTCTTCCGATCTCGTAACGNNNNNNNNNTTTTTTTTTTTTTTTTTTTTTTTTTTTTTTVN	G2	/5Biosg/ACACTCTTTCCCTACACGACGCTCTTCCGATCTGCAATCANNNNNNNNNTTTTTTTTTTTTTTTTTTTTTTTTTTTTTTVN
C3	/5Biosg/ACACTCTTTCCCTACACGACGCTCTTCCGATCTGACAGAGNNNNNNNNNTTTTTTTTTTTTTTTTTTTTTTTTTTTTTTVN	G3	/5Biosg/ACACTCTTTCCCTACACGACGCTCTTCCGATCTAGGTGTTNNNNNNNNNTTTTTTTTTTTTTTTTTTTTTTTTTTTTTTVN
C4	/5Biosg/ACACTCTTTCCCTACACGACGCTCTTCCGATCTAAAGAGCNNNNNNNNNTTTTTTTTTTTTTTTTTTTTTTTTTTTTTTVN	G4	/5Biosg/ACACTCTTTCCCTACACGACGCTCTTCCGATCTCACGCTANNNNNNNNNTTTTTTTTTTTTTTTTTTTTTTTTTTTTTTVN
C5	/5Biosg/ACACTCTTTCCCTACACGACGCTCTTCCGATCTGATTTCCNNNNNNNNNTTTTTTTTTTTTTTTTTTTTTTTTTTTTTTVN	G5	/5Biosg/ACACTCTTTCCCTACACGACGCTCTTCCGATCTGACGATCNNNNNNNNNTTTTTTTTTTTTTTTTTTTTTTTTTTTTTTVN
C6	/5Biosg/ACACTCTTTCCCTACACGACGCTCTTCCGATCTTTCACAGNNNNNNNNNTTTTTTTTTTTTTTTTTTTTTTTTTTTTTTVN	G6	/5Biosg/ACACTCTTTCCCTACACGACGCTCTTCCGATCTGACTCGANNNNNNNNNTTTTTTTTTTTTTTTTTTTTTTTTTTTTTTVN
C7	/5Biosg/ACACTCTTTCCCTACACGACGCTCTTCCGATCTGAACCTANNNNNNNNNTTTTTTTTTTTTTTTTTTTTTTTTTTTTTTVN	G7	/5Biosg/ACACTCTTTCCCTACACGACGCTCTTCCGATCTCGCTACTNNNNNNNNNTTTTTTTTTTTTTTTTTTTTTTTTTTTTTTVN
C8	/5Biosg/ACACTCTTTCCCTACACGACGCTCTTCCGATCTTTGGATCNNNNNNNNNTTTTTTTTTTTTTTTTTTTTTTTTTTTTTTVN	G8	/5Biosg/ACACTCTTTCCCTACACGACGCTCTTCCGATCTCGTGCAANNNNNNNNNTTTTTTTTTTTTTTTTTTTTTTTTTTTTTTVN
C9	/5Biosg/ACACTCTTTCCCTACACGACGCTCTTCCGATCTTCTCACTNNNNNNNNNTTTTTTTTTTTTTTTTTTTTTTTTTTTTTTVN	G9	/5Biosg/ACACTCTTTCCCTACACGACGCTCTTCCGATCTGTGTAGANNNNNNNNNTTTTTTTTTTTTTTTTTTTTTTTTTTTTTTVN
C10	/5Biosg/ACACTCTTTCCCTACACGACGCTCTTCCGATCTGAGGGAANNNNNNNNNTTTTTTTTTTTTTTTTTTTTTTTTTTTTTTVN	G10	/5Biosg/ACACTCTTTCCCTACACGACGCTCTTCCGATCTACGTAGGNNNNNNNNNTTTTTTTTTTTTTTTTTTTTTTTTTTTTTTVN
C11	/5Biosg/ACACTCTTTCCCTACACGACGCTCTTCCGATCTGTGGCATNNNNNNNNNTTTTTTTTTTTTTTTTTTTTTTTTTTTTTTVN	G11	/5Biosg/ACACTCTTTCCCTACACGACGCTCTTCCGATCTCGTTGGANNNNNNNNNTTTTTTTTTTTTTTTTTTTTTTTTTTTTTTVN
C12	/5Biosg/ACACTCTTTCCCTACACGACGCTCTTCCGATCTGGAAGATNNNNNNNNNTTTTTTTTTTTTTTTTTTTTTTTTTTTTTTVN	G12	/5Biosg/ACACTCTTTCCCTACACGACGCTCTTCCGATCTTTAGGAGNNNNNNNNNTTTTTTTTTTTTTTTTTTTTTTTTTTTTTTVN
D1	/5Biosg/ACACTCTTTCCCTACACGACGCTCTTCCGATCTTACATCGNNNNNNNNNTTTTTTTTTTTTTTTTTTTTTTTTTTTTTTVN	H1	/5Biosg/ACACTCTTTCCCTACACGACGCTCTTCCGATCTTGTGAAGNNNNNNNNNTTTTTTTTTTTTTTTTTTTTTTTTTTTTTTVN
D2	/5Biosg/ACACTCTTTCCCTACACGACGCTCTTCCGATCTTCTCGTGNNNNNNNNNTTTTTTTTTTTTTTTTTTTTTTTTTTTTTTVN	H2	/5Biosg/ACACTCTTTCCCTACACGACGCTCTTCCGATCTTGCTCCANNNNNNNNNTTTTTTTTTTTTTTTTTTTTTTTTTTTTTTVN
D3	/5Biosg/ACACTCTTTCCCTACACGACGCTCTTCCGATCTCATCGAGNNNNNNNNNTTTTTTTTTTTTTTTTTTTTTTTTTTTTTTVN	H3	/5Biosg/ACACTCTTTCCCTACACGACGCTCTTCCGATCTGGCATGANNNNNNNNNTTTTTTTTTTTTTTTTTTTTTTTTTTTTTTVN
D4	/5Biosg/ACACTCTTTCCCTACACGACGCTCTTCCGATCTGGGATCTNNNNNNNNNTTTTTTTTTTTTTTTTTTTTTTTTTTTTTTVN	H4	/5Biosg/ACACTCTTTCCCTACACGACGCTCTTCCGATCTCGTGTTCNNNNNNNNNTTTTTTTTTTTTTTTTTTTTTTTTTTTTTTVN
D5	/5Biosg/ACACTCTTTCCCTACACGACGCTCTTCCGATCTCAGTCATNNNNNNNNNTTTTTTTTTTTTTTTTTTTTTTTTTTTTTTVN	H5	/5Biosg/ACACTCTTTCCCTACACGACGCTCTTCCGATCTAAGGAAGNNNNNNNNNTTTTTTTTTTTTTTTTTTTTTTTTTTTTTTVN
D6	/5Biosg/ACACTCTTTCCCTACACGACGCTCTTCCGATCTATGAGGANNNNNNNNNTTTTTTTTTTTTTTTTTTTTTTTTTTTTTTVN	H6	/5Biosg/ACACTCTTTCCCTACACGACGCTCTTCCGATCTTTCTTCCNNNNNNNNNTTTTTTTTTTTTTTTTTTTTTTTTTTTTTTVN
D7	/5Biosg/ACACTCTTTCCCTACACGACGCTCTTCCGATCTCCTAAGANNNNNNNNNTTTTTTTTTTTTTTTTTTTTTTTTTTTTTTVN	H7	/5Biosg/ACACTCTTTCCCTACACGACGCTCTTCCGATCTTGGTACCNNNNNNNNNTTTTTTTTTTTTTTTTTTTTTTTTTTTTTTVN
D8	/5Biosg/ACACTCTTTCCCTACACGACGCTCTTCCGATCTCCCGAAANNNNNNNNNTTTTTTTTTTTTTTTTTTTTTTTTTTTTTTVN	H8	/5Biosg/ACACTCTTTCCCTACACGACGCTCTTCCGATCTAGAAAGGNNNNNNNNNTTTTTTTTTTTTTTTTTTTTTTTTTTTTTTVN
D9	/5Biosg/ACACTCTTTCCCTACACGACGCTCTTCCGATCTTTTGTGCNNNNNNNNNTTTTTTTTTTTTTTTTTTTTTTTTTTTTTTVN	H9	/5Biosg/ACACTCTTTCCCTACACGACGCTCTTCCGATCTACGCAACNNNNNNNNNTTTTTTTTTTTTTTTTTTTTTTTTTTTTTTVN
D10	/5Biosg/ACACTCTTTCCCTACACGACGCTCTTCCGATCTTATCCGTNNNNNNNNNTTTTTTTTTTTTTTTTTTTTTTTTTTTTTTVN	H10	/5Biosg/ACACTCTTTCCCTACACGACGCTCTTCCGATCTAGTCGGTNNNNNNNNNTTTTTTTTTTTTTTTTTTTTTTTTTTTTTTVN
D11	/5Biosg/ACACTCTTTCCCTACACGACGCTCTTCCGATCTCCTTCACNNNNNNNNNTTTTTTTTTTTTTTTTTTTTTTTTTTTTTTVN	H11	/5Biosg/ACACTCTTTCCCTACACGACGCTCTTCCGATCTTCCCTAANNNNNNNNNTTTTTTTTTTTTTTTTTTTTTTTTTTTTTTVN
D12	/5Biosg/ACACTCTTTCCCTACACGACGCTCTTCCGATCTCATAGCANNNNNNNNNTTTTTTTTTTTTTTTTTTTTTTTTTTTTTTVN	H12	/5Biosg/ACACTCTTTCCCTACACGACGCTCTTCCGATCTCAAACGGNNNNNNNNNTTTTTTTTTTTTTTTTTTTTTTTTTTTTTTVN

Supplementary Table 6. tSCRB Barcoded Oligo-dT Primer Plate v3

/5Biosg/ACACTCTTTCCCTACACGACGCTCTTCCGATCTJ(7)N(9)T(30)VN

/5Biosg/ denotes a 5' biotin; J denotes a nucleic acid part of the cellular barcode; N denotes a random nucleic acid part of the unique molecular identifier; V denotes A or C or G; IDT Ultramer Plate; Standard desalting

A1	/5Biosg/ACACTCTTTCCCTACACGACGCTCTTCCGATCTAACTTGGNNNNNNNNNTTTTTTTTTTTTTTTTTTTTTTTTTTTTTTVN	E1	/5Biosg/ACACTCTTTCCCTACACGACGCTCTTCCGATCTAGGTCAGNNNNNNNNNTTTTTTTTTTTTTTTTTTTTTTTTTTTTTTVN
A2	/5Biosg/ACACTCTTTCCCTACACGACGCTCTTCCGATCTCATCTGCNNNNNNNNNTTTTTTTTTTTTTTTTTTTTTTTTTTTTTTVN	E2	/5Biosg/ACACTCTTTCCCTACACGACGCTCTTCCGATCTCTACCGANNNNNNNNNTTTTTTTTTTTTTTTTTTTTTTTTTTTTTTVN
A3	/5Biosg/ACACTCTTTCCCTACACGACGCTCTTCCGATCTAGTCACANNNNNNNNNTTTTTTTTTTTTTTTTTTTTTTTTTTTTTTVN	E3	/5Biosg/ACACTCTTTCCCTACACGACGCTCTTCCGATCTTGTTCTGNNNNNNNNNTTTTTTTTTTTTTTTTTTTTTTTTTTTTTTVN
A4	/5Biosg/ACACTCTTTCCCTACACGACGCTCTTCCGATCTGCTGCTTNNNNNNNNNTTTTTTTTTTTTTTTTTTTTTTTTTTTTTTVN	E4	/5Biosg/ACACTCTTTCCCTACACGACGCTCTTCCGATCTACCATTCNNNNNNNNNTTTTTTTTTTTTTTTTTTTTTTTTTTTTTTVN
A5	/5Biosg/ACACTCTTTCCCTACACGACGCTCTTCCGATCTACTGTTGNNNNNNNNNTTTTTTTTTTTTTTTTTTTTTTTTTTTTTTVN	E5	/5Biosg/ACACTCTTTCCCTACACGACGCTCTTCCGATCTCCCTCTTNNNNNNNNNTTTTTTTTTTTTTTTTTTTTTTTTTTTTTTVN
A6	/5Biosg/ACACTCTTTCCCTACACGACGCTCTTCCGATCTACACTGANNNNNNNNNTTTTTTTTTTTTTTTTTTTTTTTTTTTTTTVN	E6	/5Biosg/ACACTCTTTCCCTACACGACGCTCTTCCGATCTGGACACTNNNNNNNNNTTTTTTTTTTTTTTTTTTTTTTTTTTTTTTVN
A7	/5Biosg/ACACTCTTTCCCTACACGACGCTCTTCCGATCTTGACGAANNNNNNNNNTTTTTTTTTTTTTTTTTTTTTTTTTTTTTTVN	E7	/5Biosg/ACACTCTTTCCCTACACGACGCTCTTCCGATCTATAGCCGNNNNNNNNNTTTTTTTTTTTTTTTTTTTTTTTTTTTTTTVN
A8	/5Biosg/ACACTCTTTCCCTACACGACGCTCTTCCGATCTTGCACGTNNNNNNNNNTTTTTTTTTTTTTTTTTTTTTTTTTTTTTTVN	E8	/5Biosg/ACACTCTTTCCCTACACGACGCTCTTCCGATCTAGTTCGCNNNNNNNNNTTTTTTTTTTTTTTTTTTTTTTTTTTTTTTVN
A9	/5Biosg/ACACTCTTTCCCTACACGACGCTCTTCCGATCTCACAAGCNNNNNNNNNTTTTTTTTTTTTTTTTTTTTTTTTTTTTTTVN	E9	/5Biosg/ACACTCTTTCCCTACACGACGCTCTTCCGATCTGAGTAACNNNNNNNNNTTTTTTTTTTTTTTTTTTTTTTTTTTTTTTVN
A10	/5Biosg/ACACTCTTTCCCTACACGACGCTCTTCCGATCTGTATGACNNNNNNNNNTTTTTTTTTTTTTTTTTTTTTTTTTTTTTTVN	E10	/5Biosg/ACACTCTTTCCCTACACGACGCTCTTCCGATCTCCGATAGNNNNNNNNNTTTTTTTTTTTTTTTTTTTTTTTTTTTTTTVN
A11	/5Biosg/ACACTCTTTCCCTACACGACGCTCTTCCGATCTAGAGCTCNNNNNNNNNTTTTTTTTTTTTTTTTTTTTTTTTTTTTTTVN	E11	/5Biosg/ACACTCTTTCCCTACACGACGCTCTTCCGATCTTGAAGCCNNNNNNNNNTTTTTTTTTTTTTTTTTTTTTTTTTTTTTTVN
A12	/5Biosg/ACACTCTTTCCCTACACGACGCTCTTCCGATCTCTAAGGTNNNNNNNNNTTTTTTTTTTTTTTTTTTTTTTTTTTTTTTVN	E12	/5Biosg/ACACTCTTTCCCTACACGACGCTCTTCCGATCTCCTGGTANNNNNNNNNTTTTTTTTTTTTTTTTTTTTTTTTTTTTTTVN
B1	/5Biosg/ACACTCTTTCCCTACACGACGCTCTTCCGATCTAAATGCGNNNNNNNNNTTTTTTTTTTTTTTTTTTTTTTTTTTTTTTVN	F1	/5Biosg/ACACTCTTTCCCTACACGACGCTCTTCCGATCTCAAGACTNNNNNNNNNTTTTTTTTTTTTTTTTTTTTTTTTTTTTTTVN
B2	/5Biosg/ACACTCTTTCCCTACACGACGCTCTTCCGATCTGAAGCGTNNNNNNNNNTTTTTTTTTTTTTTTTTTTTTTTTTTTTTTVN	F2	/5Biosg/ACACTCTTTCCCTACACGACGCTCTTCCGATCTACGGGATNNNNNNNNNTTTTTTTTTTTTTTTTTTTTTTTTTTTTTTVN
B3	/5Biosg/ACACTCTTTCCCTACACGACGCTCTTCCGATCTGCCATATNNNNNNNNNTTTTTTTTTTTTTTTTTTTTTTTTTTTTTTVN	F3	/5Biosg/ACACTCTTTCCCTACACGACGCTCTTCCGATCTAACGGTTNNNNNNNNNTTTTTTTTTTTTTTTTTTTTTTTTTTTTTTVN
B4	/5Biosg/ACACTCTTTCCCTACACGACGCTCTTCCGATCTATGCATGNNNNNNNNNTTTTTTTTTTTTTTTTTTTTTTTTTTTTTTVN	F4	/5Biosg/ACACTCTTTCCCTACACGACGCTCTTCCGATCTTACGCCTNNNNNNNNNTTTTTTTTTTTTTTTTTTTTTTTTTTTTTTVN
B5	/5Biosg/ACACTCTTTCCCTACACGACGCTCTTCCGATCTTCGGACANNNNNNNNNTTTTTTTTTTTTTTTTTTTTTTTTTTTTTTVN	F5	/5Biosg/ACACTCTTTCCCTACACGACGCTCTTCCGATCTCACCATGNNNNNNNNNTTTTTTTTTTTTTTTTTTTTTTTTTTTTTTVN
B6	/5Biosg/ACACTCTTTCCCTACACGACGCTCTTCCGATCTGATCCACNNNNNNNNNTTTTTTTTTTTTTTTTTTTTTTTTTTTTTTVN	F6	/5Biosg/ACACTCTTTCCCTACACGACGCTCTTCCGATCTCGAGGTTNNNNNNNNNTTTTTTTTTTTTTTTTTTTTTTTTTTTTTTVN
B7	/5Biosg/ACACTCTTTCCCTACACGACGCTCTTCCGATCTCGGTATANNNNNNNNNTTTTTTTTTTTTTTTTTTTTTTTTTTTTTTVN	F7	/5Biosg/ACACTCTTTCCCTACACGACGCTCTTCCGATCTTTCTGGANNNNNNNNNTTTTTTTTTTTTTTTTTTTTTTTTTTTTTTVN
B8	/5Biosg/ACACTCTTTCCCTACACGACGCTCTTCCGATCTACAGGCANNNNNNNNNTTTTTTTTTTTTTTTTTTTTTTTTTTTTTTVN	F8	/5Biosg/ACACTCTTTCCCTACACGACGCTCTTCCGATCTGGATTCGNNNNNNNNNTTTTTTTTTTTTTTTTTTTTTTTTTTTTTTVN
B9	/5Biosg/ACACTCTTTCCCTACACGACGCTCTTCCGATCTGTAATGGNNNNNNNNNTTTTTTTTTTTTTTTTTTTTTTTTTTTTTTVN	F9	/5Biosg/ACACTCTTTCCCTACACGACGCTCTTCCGATCTGTTTGCGNNNNNNNNNTTTTTTTTTTTTTTTTTTTTTTTTTTTTTTVN
B10	/5Biosg/ACACTCTTTCCCTACACGACGCTCTTCCGATCTAGGCTTANNNNNNNNNTTTTTTTTTTTTTTTTTTTTTTTTTTTTTTVN	F10	/5Biosg/ACACTCTTTCCCTACACGACGCTCTTCCGATCTCCACACANNNNNNNNNTTTTTTTTTTTTTTTTTTTTTTTTTTTTTTVN
B11	/5Biosg/ACACTCTTTCCCTACACGACGCTCTTCCGATCTGGGCAAANNNNNNNNNTTTTTTTTTTTTTTTTTTTTTTTTTTTTTTVN	F11	/5Biosg/ACACTCTTTCCCTACACGACGCTCTTCCGATCTCCTTTGGNNNNNNNNNTTTTTTTTTTTTTTTTTTTTTTTTTTTTTTVN
B12	/5Biosg/ACACTCTTTCCCTACACGACGCTCTTCCGATCTCTGAGACNNNNNNNNNTTTTTTTTTTTTTTTTTTTTTTTTTTTTTTVN	F12	/5Biosg/ACACTCTTTCCCTACACGACGCTCTTCCGATCTACTACGTNNNNNNNNNTTTTTTTTTTTTTTTTTTTTTTTTTTTTTTVN
C1	/5Biosg/ACACTCTTTCCCTACACGACGCTCTTCCGATCTATACACCNNNNNNNNNTTTTTTTTTTTTTTTTTTTTTTTTTTTTTTVN	G1	/5Biosg/ACACTCTTTCCCTACACGACGCTCTTCCGATCTTCAACTCNNNNNNNNNTTTTTTTTTTTTTTTTTTTTTTTTTTTTTTVN
C2	/5Biosg/ACACTCTTTCCCTACACGACGCTCTTCCGATCTCGTAACGNNNNNNNNNTTTTTTTTTTTTTTTTTTTTTTTTTTTTTTVN	G2	/5Biosg/ACACTCTTTCCCTACACGACGCTCTTCCGATCTGCAATCANNNNNNNNNTTTTTTTTTTTTTTTTTTTTTTTTTTTTTTVN
C3	/5Biosg/ACACTCTTTCCCTACACGACGCTCTTCCGATCTGACAGAGNNNNNNNNNTTTTTTTTTTTTTTTTTTTTTTTTTTTTTTVN	G3	/5Biosg/ACACTCTTTCCCTACACGACGCTCTTCCGATCTAGGTGTTNNNNNNNNNTTTTTTTTTTTTTTTTTTTTTTTTTTTTTTVN
C4	/5Biosg/ACACTCTTTCCCTACACGACGCTCTTCCGATCTAAAGAGCNNNNNNNNNTTTTTTTTTTTTTTTTTTTTTTTTTTTTTTVN	G4	/5Biosg/ACACTCTTTCCCTACACGACGCTCTTCCGATCTCACGCTANNNNNNNNNTTTTTTTTTTTTTTTTTTTTTTTTTTTTTTVN
C5	/5Biosg/ACACTCTTTCCCTACACGACGCTCTTCCGATCTGATTTCCNNNNNNNNNTTTTTTTTTTTTTTTTTTTTTTTTTTTTTTVN	G5	/5Biosg/ACACTCTTTCCCTACACGACGCTCTTCCGATCTGACGATCNNNNNNNNNTTTTTTTTTTTTTTTTTTTTTTTTTTTTTTVN
C6	/5Biosg/ACACTCTTTCCCTACACGACGCTCTTCCGATCTTTCACAGNNNNNNNNNTTTTTTTTTTTTTTTTTTTTTTTTTTTTTTVN	G6	/5Biosg/ACACTCTTTCCCTACACGACGCTCTTCCGATCTGACTCGANNNNNNNNNTTTTTTTTTTTTTTTTTTTTTTTTTTTTTTVN
C7	/5Biosg/ACACTCTTTCCCTACACGACGCTCTTCCGATCTGAACCTANNNNNNNNNTTTTTTTTTTTTTTTTTTTTTTTTTTTTTTVN	G7	/5Biosg/ACACTCTTTCCCTACACGACGCTCTTCCGATCTCGCTACTNNNNNNNNNTTTTTTTTTTTTTTTTTTTTTTTTTTTTTTVN
C8	/5Biosg/ACACTCTTTCCCTACACGACGCTCTTCCGATCTTTGGATCNNNNNNNNNTTTTTTTTTTTTTTTTTTTTTTTTTTTTTTVN	G8	/5Biosg/ACACTCTTTCCCTACACGACGCTCTTCCGATCTCGTGCAANNNNNNNNNTTTTTTTTTTTTTTTTTTTTTTTTTTTTTTVN
C9	/5Biosg/ACACTCTTTCCCTACACGACGCTCTTCCGATCTTCTCACTNNNNNNNNNTTTTTTTTTTTTTTTTTTTTTTTTTTTTTTVN	G9	/5Biosg/ACACTCTTTCCCTACACGACGCTCTTCCGATCTGTGTAGANNNNNNNNNTTTTTTTTTTTTTTTTTTTTTTTTTTTTTTVN
C10	/5Biosg/ACACTCTTTCCCTACACGACGCTCTTCCGATCTGAGGGAANNNNNNNNNTTTTTTTTTTTTTTTTTTTTTTTTTTTTTTVN	G10	/5Biosg/ACACTCTTTCCCTACACGACGCTCTTCCGATCTACGTAGGNNNNNNNNNTTTTTTTTTTTTTTTTTTTTTTTTTTTTTTVN
C11	/5Biosg/ACACTCTTTCCCTACACGACGCTCTTCCGATCTGTGGCATNNNNNNNNNTTTTTTTTTTTTTTTTTTTTTTTTTTTTTTVN	G11	/5Biosg/ACACTCTTTCCCTACACGACGCTCTTCCGATCTCGTTGGANNNNNNNNNTTTTTTTTTTTTTTTTTTTTTTTTTTTTTTVN
C12	/5Biosg/ACACTCTTTCCCTACACGACGCTCTTCCGATCTGGAAGATNNNNNNNNNTTTTTTTTTTTTTTTTTTTTTTTTTTTTTTVN	G12	/5Biosg/ACACTCTTTCCCTACACGACGCTCTTCCGATCTTTAGGAGNNNNNNNNNTTTTTTTTTTTTTTTTTTTTTTTTTTTTTTVN
D1	/5Biosg/ACACTCTTTCCCTACACGACGCTCTTCCGATCTTACATCGNNNNNNNNNTTTTTTTTTTTTTTTTTTTTTTTTTTTTTTVN	H1	/5Biosg/ACACTCTTTCCCTACACGACGCTCTTCCGATCTTGTGAAGNNNNNNNNNTTTTTTTTTTTTTTTTTTTTTTTTTTTTTTVN
D2	/5Biosg/ACACTCTTTCCCTACACGACGCTCTTCCGATCTTCTCGTGNNNNNNNNNTTTTTTTTTTTTTTTTTTTTTTTTTTTTTTVN	H2	/5Biosg/ACACTCTTTCCCTACACGACGCTCTTCCGATCTTGCTCCANNNNNNNNNTTTTTTTTTTTTTTTTTTTTTTTTTTTTTTVN
D3	/5Biosg/ACACTCTTTCCCTACACGACGCTCTTCCGATCTCATCGAGNNNNNNNNNTTTTTTTTTTTTTTTTTTTTTTTTTTTTTTVN	H3	/5Biosg/ACACTCTTTCCCTACACGACGCTCTTCCGATCTGGCATGANNNNNNNNNTTTTTTTTTTTTTTTTTTTTTTTTTTTTTTVN
D4	/5Biosg/ACACTCTTTCCCTACACGACGCTCTTCCGATCTGGGATCTNNNNNNNNNTTTTTTTTTTTTTTTTTTTTTTTTTTTTTTVN	H4	/5Biosg/ACACTCTTTCCCTACACGACGCTCTTCCGATCTCGTGTTCNNNNNNNNNTTTTTTTTTTTTTTTTTTTTTTTTTTTTTTVN
D5	/5Biosg/ACACTCTTTCCCTACACGACGCTCTTCCGATCTCAGTCATNNNNNNNNNTTTTTTTTTTTTTTTTTTTTTTTTTTTTTTVN	H5	/5Biosg/ACACTCTTTCCCTACACGACGCTCTTCCGATCTAAGGAAGNNNNNNNNNTTTTTTTTTTTTTTTTTTTTTTTTTTTTTTVN
D6	/5Biosg/ACACTCTTTCCCTACACGACGCTCTTCCGATCTATGAGGANNNNNNNNNTTTTTTTTTTTTTTTTTTTTTTTTTTTTTTVN	H6	/5Biosg/ACACTCTTTCCCTACACGACGCTCTTCCGATCTTTCTTCCNNNNNNNNNTTTTTTTTTTTTTTTTTTTTTTTTTTTTTTVN
D7	/5Biosg/ACACTCTTTCCCTACACGACGCTCTTCCGATCTCCTAAGANNNNNNNNNTTTTTTTTTTTTTTTTTTTTTTTTTTTTTTVN	H7	/5Biosg/ACACTCTTTCCCTACACGACGCTCTTCCGATCTTGGTACCNNNNNNNNNTTTTTTTTTTTTTTTTTTTTTTTTTTTTTTVN
D8	/5Biosg/ACACTCTTTCCCTACACGACGCTCTTCCGATCTCCCGAAANNNNNNNNNTTTTTTTTTTTTTTTTTTTTTTTTTTTTTTVN	H8	/5Biosg/ACACTCTTTCCCTACACGACGCTCTTCCGATCTAGAAAGGNNNNNNNNNTTTTTTTTTTTTTTTTTTTTTTTTTTTTTTVN
D9	/5Biosg/ACACTCTTTCCCTACACGACGCTCTTCCGATCTTTTGTGCNNNNNNNNNTTTTTTTTTTTTTTTTTTTTTTTTTTTTTTVN	H9	/5Biosg/ACACTCTTTCCCTACACGACGCTCTTCCGATCTACGCAACNNNNNNNNNTTTTTTTTTTTTTTTTTTTTTTTTTTTTTTVN
D10	/5Biosg/ACACTCTTTCCCTACACGACGCTCTTCCGATCTTATCCGTNNNNNNNNNTTTTTTTTTTTTTTTTTTTTTTTTTTTTTTVN	H10	/5Biosg/ACACTCTTTCCCTACACGACGCTCTTCCGATCTAGTCGGTNNNNNNNNNTTTTTTTTTTTTTTTTTTTTTTTTTTTTTTVN
D11	/5Biosg/ACACTCTTTCCCTACACGACGCTCTTCCGATCTCCTTCACNNNNNNNNNTTTTTTTTTTTTTTTTTTTTTTTTTTTTTTVN	H11	/5Biosg/ACACTCTTTCCCTACACGACGCTCTTCCGATCTGGTTATCNNNNNNNNNTTTTTTTTTTTTTTTTTTTTTTTTTTTTTTVN
D12	/5Biosg/ACACTCTTTCCCTACACGACGCTCTTCCGATCTCATAGCANNNNNNNNNTTTTTTTTTTTTTTTTTTTTTTTTTTTTTTVN	H12	/5Biosg/ACACTCTTTCCCTACACGACGCTCTTCCGATCTCAAACGGNNNNNNNNNTTTTTTTTTTTTTTTTTTTTTTTTTTTTTTVN
