# Supplementary material for: Tailoring the resolution of single-cell RNA sequencing for primary cytotoxic T cells
Source: Nat Commun. 2021 Jan 25;12:569. doi: 10.1038/s41467-020-20751-7 (PMC7835213; doi:10.1038/s41467-020-20751-7)
Supplement: Supplementary file 3 — Reporting Summary [file 41467_2020_20751_MOESM3_ESM.pdf]

nature research

Corresponding author(s): Kristiyan Kanev, Dietmar Zehn

Last updated by author(s): Dec 13, 2020

Reporting Summary

Nature Research wishes to improve the reproducibility of the work that we publish. This form provides structure for consistency and transparency in reporting. For further information on Nature Research policies, see our Editorial Policies and the Editorial Policy Checklist.

Statistics

For all statistical analyses, confirm that the following items are present in the figure legend, table legend, main text, or Methods section.

n/a	Confirmed	
☐	☒	The exact sample size (*n*) for each experimental group/condition, given as a discrete number and unit of measurement
☐	☒	A statement on whether measurements were taken from distinct samples or whether the same sample was measured repeatedly
☒	☐	The statistical test(s) used AND whether they are one- or two-sided<br>*Only common tests should be described solely by name; describe more complex techniques in the Methods section.*
☐	☒	A description of all covariates tested
☒	☐	A description of any assumptions or corrections, such as tests of normality and adjustment for multiple comparisons
☐	☒	A full description of the statistical parameters including central tendency (e.g. means) or other basic estimates (e.g. regression coefficient) AND variation (e.g. standard deviation) or associated estimates of uncertainty (e.g. confidence intervals)
☒	☐	For null hypothesis testing, the test statistic (e.g. *F*, *t*, *r*) with confidence intervals, effect sizes, degrees of freedom and *P* value noted<br>*Give P values as exact values whenever suitable.*
☒	☐	For Bayesian analysis, information on the choice of priors and Markov chain Monte Carlo settings
☒	☐	For hierarchical and complex designs, identification of the appropriate level for tests and full reporting of outcomes
☒	☐	Estimates of effect sizes (e.g. Cohen's *d*, Pearson's *r*), indicating how they were calculated

Our web collection on statistics for biologists contains articles on many of the points above.

Software and code

Policy information about availability of computer code

Data collection

DropSeqPipe v0.4 was used for raw data processing (https://hoohm.github.io/dropSeqPipe/)

Cutadapt v1.16 was used for trimming (https://cutadapt.readthedocs.io/en/v1.16/guide.html).

BBMap v38.22 was used to discard reads with a missing pair (https://jgi.doe.gov/data-and-tools/bbtools/bb-tools-user-guide/bbmap-guide/).

STAR v2.5.3a 25 was used for read mapping (https://github.com/alexdobin/STAR).

Dropseq_tools v1.13 was used for demultiplexing and file manipulation (https://github.com/broadinstitute/Drop-seq/releases).

Data analysis

Single-cell data analysis:

> M3Drop v1.12.0 was used to identify significantly variable genes for use in downstream cell clustering (https://www.bioconductor.org/packages/release/bioc/html/M3Drop.html)

> Seurat v 2.3.4 was used for further single-cell data visualization and clustering (https://satijalab.org/seurat/)

Statistical analysis:

> GraphPad Prism 6.0

FACS data analysis:

> FlowJo™ Software Version 9 and 10

For manuscripts utilizing custom algorithms or software that are central to the research but not yet described in published literature, software must be made available to editors and reviewers. We strongly encourage code deposition in a community repository (e.g. GitHub). See the Nature Research guidelines for submitting code & software for further information.

Data

Policy information about availability of data

All manuscripts must include a data availability statement. This statement should provide the following information, where applicable:
- Accession codes, unique identifiers, or web links for publicly available datasets
- A list of figures that have associated raw data
- A description of any restrictions on data availability

Data generated during the study are available in a public repository GSE163089 [https://www.ncbi.nlm.nih.gov/geo/query/acc.cgi?acc=GSE163089]

Field-specific reporting

Please select the one below that is the best fit for your research. If you are not sure, read the appropriate sections before making your selection.

- [x] Life sciences
- [ ] Behavioural & social sciences
- [ ] Ecological, evolutionary & environmental sciences

For a reference copy of the document with all sections, see nature.com/documents/nr-reporting-summary-flat.pdf

Life sciences study design

All studies must disclose on these points even when the disclosure is negative.

Sample size	For the animal studies, power analysis was used to determine the number of animals per group to reach a confidence limit of 95%. The infection models used in this study are well established and yield very low variance. Owing to this low variance, statistical power is achieved with 3-4 animals per group. To account for potential misinjection of the P14 transgenic cells, 5 animals per group were approved to be used in these experiments by the regional veterinary authority "Regierung of Oberbayern". Thus, all experimental groups in this study contained 5 animals.
Data exclusions	We did not exclude individual results from the presented data.
Replication	The Drop-seq human and mouse lymphocyte mixing experiment was repeated three times. The baseline Drop-seq protocol and each separate Drop-seq optimization with naïve P14s were repeated once. The final tDrop-seq protocol with P14 T-cell recovered at day 8 post LCMV Arm infection was repeated two times. The baseline SCBR-seq protocol and each separate SCBR-seq optimization were repeated once. The final tSCRB-seq protocol with P14 T-cell recovered at day 8 post LCMV Arm infection was performed with ten plates processed on three different days, each plate representing an individual replicate.
Randomization	Mouse study groups were designed by randomly including entire mouse cages.
Blinding	No-blinded experiments. The binding was due the use of animals from the same sex, age and inbred strain.

Reporting for specific materials, systems and methods

We require information from authors about some types of materials, experimental systems and methods used in many studies. Here, indicate whether each material, system or method listed is relevant to your study. If you are not sure if a list item applies to your research, read the appropriate section before selecting a response.

Materials & experimental systems

n/a	Involved in the study	
[ ]	[x]	Antibodies
[x]	[ ]	Eukaryotic cell lines
[x]	[ ]	Palaeontology and archaeology
[ ]	[x]	Animals and other organisms
[x]	[ ]	Human research participants
[x]	[ ]	Clinical data
[x]	[ ]	Dual use research of concern

Methods

n/a	Involved in the study	
[x]	[ ]	ChIP-seq
[ ]	[x]	Flow cytometry
[x]	[ ]	MRI-based neuroimaging

Antibodies

Antibodies used	We used the following mouse specific antibodies in our studies:<br>Supplier' 'Antigen' 'clone' 'label' 'lot number' 'catalog number' 'Dilution<br>>Biolegend' 'CD8' '53-6.7' 'PerCP/Cy5.5' 'B231083' '100734' '1-400<br>>Biolegend' 'CD8' '53-6.7' 'APC' 'B256876' '100712' '1-400<br>>Biolegend' 'CD4' 'RM4-4' 'FITC 'B247298' '116004' '1-400<br>>eBioscience' 'TCR V alpha 2' 'B20.1' 'PE' '1982690' '12-5812-82 '1-400<br>>eBioscience' 'CD45.1' 'A20' 'PE' '4300554' '12-0453-83' '1-400<br>>eBioscience' 'CD45.1' 'A20' 'Biotin' '4276799' '13-0453-85' '1-200<br>>eBioscience' 'CD45.2' '104' 'eFluor® 450' '4344415' '48-0454-82' '1-400
Validation	All antibodies were validated as part of the quality control process of the manufacture.<br>Manufacturer validation of CD8 clone 53-6.7 - PerCP/Cyanine5.5: C57BL/6 mouse splenocytes were stained with CD3e FITC and CD8a (clone 53-6.7) PerCP/Cyanine5.5, or Rat IgG2a, PerCP/Cyanine5.5 isotype control.<br>Manufacturer validation of CD8 clone 53-6.7 - APC: C57BL/6 mouse splenocytes were stained with CD8 (clone 53-6.7) APC or rat IgG2a, κ APC isotype control.<br>Manufacturer validation of CD4 clone RM4-4 - FITC: C57BL/6 mouse splenocytes were stained with CD3e FITC and CD4 (clone RM4-4) FITC, or rat IgG2b, FITC isotype control.<br>Manufacturer validation of TCR V alpha 2 clone B20.1 - PE: Staining of C57Bl/6 splenocytes with Anti-Mouse CD4 FITC and Anti-Mouse CD8a FITC and 0.25 µg of Rat IgG2a K Isotype Control PE or 0.25 µg of Anti-Mouse V alpha 2 TCR PE.<br>Manufacturer validation of CD45.1 clone A20 - PE: Staining of C57BL/6 or SJL mouse splenocytes with 0.25 µg of Mouse IgG2a kappa Isotype Control, PE or 0.25 µg of CD45.1 clone A20 – PE.<br>Manufacturer validation of CD45.1 clone A20 - Biotin: Staining of SJL mouse splenocytes with 0.125 µg of Mouse IgG2a kappa Isotype Control Biotin or 0.125 µg of Anti-Mouse CD45.1 Biotin followed by Streptavidin PE<br>Manufacturer validation of CD45.2 clone 104- eFluor 450: Staining of SJL and BALB/c splenocytes with 0.25 µg of Mouse IgG2a K Isotype Control eFluor® 450 or 0.25 µg of Anti-Mouse CD45-2 eFluor® 450.<br>Additionally, all antibodies were validated in an internal quality control process using lymphocytes recovered from congenic animals. All antibodies stained specifically the expected populations.

Animals and other organisms

Policy information about studies involving animals; ARRIVE guidelines recommended for reporting animal research

Laboratory animals	Tcrα Knockout/ T Cell Receptor Transgenic P14 mice on C57BL/6 background (B6.Cg-Tcratm1Mom Tg(TcrLCMV)327Sdz) were used as donors of gp33-specific P14 CD8 T-cells. The donor animals were 8-10 weeks old. The transgenic P14s were transferred into sex-matched hosts of C57BL/6J and C57BL/6N background. At the beginning of the experiments, the hosts were between 6-10 weeks of age. Male and female mice were used. Mice were maintained at a temperature 20 – 24oC, humidity 50-70 % and 12 hours light cycle with light phase beginning at 5 am and ending at 5 pm.
Wild animals	Wild animals were not involved in the study
Field-collected samples	The study did not involve sample collected from the field
Ethics oversight	The experiments were performed in compliance with institutional guidelines of the Technical University of Munich and were legally approved by the regional veterinarian authority "Regierung of Oberbayern".

Note that full information on the approval of the study protocol must also be provided in the manuscript.

Flow Cytometry

Plots

Confirm that:

- [x] The axis labels state the marker and fluorochrome used (e.g. CD4-FITC).
- [x] The axis scales are clearly visible. Include numbers along axes only for bottom left plot of group (a 'group' is an analysis of identical markers).
- [x] All plots are contour plots with outliers or pseudocolor plots.
- [x] A numerical value for number of cells or percentage (with statistics) is provided.

Methodology

Sample preparation	We used standard methods for sample preparation as indicated in the submitted supplementary methods section
Instrument	BD FACS Fusion

Software	Flow Jo Version 9 and 10
Cell population abundance	For the Drop-seq experiments the post sort purity was >98. For the SCRB-seq experiment, single-cell were sorted into individual wells of a PCR plate (single-cell sort purity).
Gating strategy	We used standard gating strategies: Live / Dead discrimination based on FSC and SSC signals, gating on the typical lymphocyte<br>population based on FSC SSC signals, doublet exclusion based on FSC-H and FSC-A comparison, back-gating tests to ensure that<br>minor sub-populations were not excluded by the gating strategy

☒ Tick this box to confirm that a figure exemplifying the gating strategy is provided in the Supplementary Information.
